# Twenty-fourth Annual Report of the Bloomingdale Asylum for the Insane

**Published:** 1845-04

**Authors:** 


*Twenty-fourth Annual Report of the Bloomingdale Asylum for the Insane.* By PLINY EARL, M. D., Physician to the Institution.

This is a very full and able Report. Our correspondent, to whom we are indebted for a notice of the annual reports of several similar Institutions, in the present number, will doubtless embrace Dr. Earl's in a future communication.

